# Hospital Meetings

**Published:** 1906-05-26

**Authors:** 


### HOSPITAL MEETINGS.

### HAMPSTEAD GENERAL HOSPITAL.

### A RECORD COLLECTION.

The biennial festival dinner of the Hampstead General Hospital took place at the Grand Hotel on Thursday evening (17th inst.), the Deputy-Mayor of Hampstead (Alderman Donald McMillan) in the chair.

#### An Example to be Followed.

This hospital, which during the course of last year moved into its new buildings, containing 62 beds, has set an example to the other general hospitals of the metropolis in that it has, in the accounts for the year 1905, kept separately the expenditure on its out-patient department. From this account it would appear that the cost of each out-patient during the year amounted to 1s. 9½d., whilst each attendance cost 6.8d.

#### "Prosperity to the Hospital."

The Chairman, in proposing "Prosperity to the Hampstead General Hospital," referred with deep regret to the sad bereavement which had prevented the Mayor of Hampstead from presiding that evening. This was the first occasion that the festival dinner had been held since the opening of the new hospital, and to-day they had a building of which Hampstead people might well be proud. The fact of having such a building brought with it great responsibility, and it rested on the citizens of Hampstead to show that they were equal to the occasion, and that, having obtained such a building, they would see that it was equipped and carried on in a thoroughly satisfactory manner. At the present time there were 33 free beds, 20 cots, and nine contributory beds (to which patients were admitted on a payment of 12s. a week). The extension of the building which was contemplated, and which would, he believed, be shortly commenced, had been rendered possible by the munificent donation of £20,000 which was promised by an anonymous donor through Sir Henry Harben. When that extension was completed there would be a further 36 free beds and 13 contributcry beds, making a total of 91 beds and 20 cots. That brought him to the financial question, the most difficult matter with which the Council of the hospital had to deal. At the end of 1905 there was a debit balance on the maintenance account of £1,300, whilst the amount necessary for 1906 was about £5,000, or a total requirement of £6,300. Towards that the average income of the hospital was only £2,000, leaving a balance to be raised in one way or another of £4,300. In conclusion he announced that the Mayor of Hampstead had handed to him a declaration of trust in connection with £1,000 in Consols to be devoted to the maintenance of the hospital.

#### The Rating of Hospitals.

Mr. Ernest Collins (Chairman of the Council) replied, and suggested the formation of a Foundation Fund, to which he hoped, from what he had heard that evening, that they would shortly receive a donation of £1,000. Referring to the rating of hospitals, he hoped an Act for the Relief of Charitable Institutions from Rating would soon be passed, as it appeared to him dishonest that money contributed to charity should, in any way at all, go towards the relief of the ratepayers.

Dr. W. Heath Strange (founder of the hospital) also responded.

#### A Record Collection.

The Secretary announced that the appeal in connection with the dinner had resulted in a collection of £3,145, by far the largest amount ever collected at the festival dinner of this hospital.

#### A Retrospect.

Sir Henry C. Burdett proposed the toast of "The Council and Medical Staff." He said that, looking back to what hospitals were forty years ago, one could hardly credit that it had been possible to make the voluntary hospitals the veritable palaces of pain which they were in the present day. He remembered the time when some of the great hospitals in London had no better accommodation for their nurses than a sort of coop let in under the stairs; whilst other nurses had to sleep in the basement, where the floors were tiled, where sometimes even there were open drains in the centre of the room, and where, it was almost unnecessary to say, sore throat and even diphtheria were constant concomitants. Then, too, the condition of the wards themselves, both in regard to cleanliness and appearance, was, to our more modern ideas, lacking in much. They all had a somewhat "workhousy" look, and the brightest of decoration was whitewash, which was not too often renewed; and when renewed the practice was, not to wash off the impurities first, but to add another coat of whitewash over the old. It was quite conceivable, therefore, that up to even twenty-five years ago and even later, it was rightly felt by the majority of people that hospitals were places which, on the whole, wise people avoided. Now, however, all that had been changed, and the difficulty which hospitals had to face in the present day was how to keep people out of their wards. The feeling had grown up that when a man or a woman fell ill the wise thing to do was to go into a hospital. That feeling they owed chiefly to the wisdom of the managers of the hospitals and to the skill of the medical profession. That was an important reason why those present should drink the toast he had to propose to them with hearty appreciation.

#### The Responsibility of the People of Hampstead.

The people of Hampstead had taken upon themselves a grave responsibility. They had enlarged their hospital, and they were committed to increasing it to 100 beds. He did not know whether they were aware of the fact that financially the management were not at present justified by the support they were receiving from the inhabitants of Hampstead in spending money, although they had it in hand, in carrying out the extensions which they were about to commence. The financial position was one of much gravity. They last year had an income of a little over £2,000, and the hospital as it was at present constituted necessitated an expenditure of £5,000 a year. That was to say the people of Hampstead would have to double their contributions—and more than double them—in order to support the present institution as it should be supported. But when they had completed all the buildings, they would require at least £7,000 a year to keep the hospital going; so that Hampstead would have to find £5,000 a year more than it did at present. The position, therefore, was a very serious one, because he knew of nothing so bad in connection with a hospital as overbuilding; for in that case they put their money out, not at interest, but into buildings; and if they did not occupy those buildings they were going to add materially to the cost of maintaining those which they did use. He therefore did most seriously hope that, so far as those present that evening were concerned they would drink that toast with a deep sense of what that act meant. It meant that they, the people of Hampstead—who occupied the healthiest site in all the metropolis, who had the finest air and the best of everything that nature could confer on the inhabitants of a great suburb, and who thereby enjoyed health and strength which was not permitted to the more crowded and less fortunate parts of the metropolis—had granted to them an opportunity of rendering praise for the blessings they enjoyed by the support they rendered to the hospital. He hoped they would all recognise their duty, and would by their voice and influence, and by giving some measure of their time, assist in securing for the Hampstead Hospital at least the greater portion of the money necessary for its support. Further, they must realise this, that by erecting that new hospital they were taking it out of the narrow ways of a small and relatively unimportant institution, and putting it upon the broad basis of one of the general hospitals of London; and in doing this they took upon themselves the further responsibility of seeing that they provided, by the appointment of an adequate honorary medical staff, for the work being carried on in a way which would be worthy of this great metropolis.

Messrs. Edmund K. Blyth and A. H. Cook responded, and the proceedings shortly afterwards terminated.

### CHELSEA HOSPITAL FOR WOMEN.

The chair was taken at the recent annual meeting of the Governors of the Chelsea Hospital for Women by the President, Lord Glenesk, who, in moving the adoption of the report, said that he did not think they had ever had such a report as this put in their hands before, showing as it did every sign of health and vigour and a very satisfactory balance. He had always had the highest hopes that this hospital would succeed, as it had done, and he had been strengthened by the knowledge of the merits of its doctors, and certainly no institution ever had a more capable and distinguished medical and surgical staff than this, who had all done honour to themselves and produced the success they now saw in the hospital. As to the nurses, it was almost needless to say what they were and how they had done their work. He had been very happy to become their President. After referring briefly to the troublous times the hospital had passed through in former years he went on to say that he could not speak too highly in praise of the members of the Council. Changes had occurred in their ranks, Mr. T. Dyer Edwards having become Chairman of the Council in his place, and they were very fortunate in having secured the very able services of Sir F. W. R. Fryer as Vice-Chairman. A member of the Council, Major-General H. Wemyss had been kind enough to hold fire-drills for the staff. The number of in-patients treated in 1905 was 787 and the number of major operations performed was 360, while the death rate was only 2 per cent., which he considered to be perfectly amazing. The work that the hospital was doing was becoming widely known by means of the patients and their friends, who were always most grateful for the kindness shown them. The Convalescent Home at St. Leonards, which was the child of their Treasurer, Mr. H. E. Wright, was also doing very good work. Their great benefactress "H. M. E.," who had given them in all, since 1894 more than £8,000, had recently died. Her name, he might tell them, was Mrs. Everard. She had preferred to give her money in her lifetime and not to leave it to be taxed afterwards. With the sums of money she had given them from time to time they had founded an Emergency Fund, which was only to be drawn upon in time of need. He was glad to say that the King's Hospital Fund had given them a larger grant last year and so had the Saturday Fund, and in every way the hospital was rising in the estimation of their fellow citizens.

Mr. H. E. Wright seconded the motion, which was adopted.

Dr. Hugh Fenton, in moving the re-election of the Council, paid a high tribute to the splendid work done by Lord Glenesk during the years he had been connected with the hospital. The staff could not do their work well and efficiently unless they were supported by a broad-minded and generous Council. The staff was always having to appeal for funds to carry out various projects. All their skill would be thwarted if they were not provided with all the most modern appliances and apparatus. They must, however, go on and not sit down and say this was the best special hospital in London, or in the world, as it was, but continue renewing damaged parts and what they now thought was perfection. As knowledge and skill advanced, they would want better things. They must not listen to the captious critic, who was always talking of hospital expenses, but never subscribed much himself. They did not want extravagance, but they did want efficiency, and they could not get that without money.

The resolution was seconded by Mrs. Shipway. Votes of thanks to the honorary medical staff and to the Chairman were subsequently adopted, and the proceedings then terminated.

### PERTH INFIRMARY REORGANISATION.

At a meeting of the Perth Infirmary directors held on May 16 it was unanimously agreed to ask Dr. Macintosh, of the Western Infirmary, Glasgow, to examine and report to the directors on the whole inside organisation of the infirmary, and to advise them what steps to take to put the institution on a more economical footing.

